# Zebrafish, a Novel Model System to Study Uremic Toxins: The Case for the Sulfur Amino Acid Lanthionine

**DOI:** 10.3390/ijms19051323

**Published:** 2018-04-29

**Authors:** Alessandra F. Perna, Evgeniya Anishchenko, Carmela Vigorito, Miriam Zacchia, Francesco Trepiccione, Salvatore D’Aniello, Diego Ingrosso

**Affiliations:** 1 First Division of Nephrology, Department of Cardiotoracic & Respiratory Sciences, University of Campania “Luigi Vanvitelli”, School of Medicine, via Pansini 5, Bldg 17, 80131 Naples, Italy; alessandra.perna@unicampania.it (A.F.P.); anishchenkoea@gmail.com (E.A.); ca.vigorito86@libero.it (C.V.); miriam.zacchia@unicampania.it (M.Z.); francesco.trepiccione@unicampania.it (F.T.); 2 Department of Precision Medicine, University of Campania “Luigi Vanvitelli”, School of Medicine, via Luigi de Crecchio 7, 80138 Naples, Italy; 3 Biogem, Contrada Camporeale, 83031 Ariano Irpino AV, Italy; 4 Biology and Evolution of Marine Organisms, Stazione Zoologica Anton Dohrn Napoli, Villa Comunale, 80121 Naples, Italy; salvatore.daniello@szn.it

**Keywords:** uremic toxin, lanthionine, uremia, dialysis, cardiovascular disease, zebrafish, glutathione

## Abstract

The non-proteinogenic amino acid lanthionine is a byproduct of hydrogen sulfide biosynthesis: the third endogenous vasodilator gas, after nitric oxide and carbon monoxide. While hydrogen sulfide is decreased in uremic patients on hemodialysis, lanthionine is increased and has been proposed as a new uremic toxin, since it is able to impair hydrogen sulfide production in hepatoma cells. To characterize lanthionine as a uremic toxin, we explored its effects during the early development of the zebrafish (*Danio rerio*), a widely used model to study the organ and tissue alterations induced by xenobiotics. Lanthionine was employed at concentrations reproducing those previously detected in uremia. Light-induced visual motor response was also studied by means of the DanioVision system. Treatment of zebrafish embryos with lanthionine determined acute phenotypical alterations, on heart organogenesis (disproportion in cardiac chambers), increased heart beating, and arrhythmia. Lanthionine also induced locomotor alterations in zebrafish embryos. Some of these effects could be counteracted by glutathione. Lanthionine exerted acute effects on transsulfuration enzymes and the expression of genes involved in inflammation and metabolic regulation, and modified microRNA expression in a way comparable with some alterations detected in uremia. Lanthionine meets the criteria for classification as a uremic toxin. Zebrafish can be successfully used to explore uremic toxin effects.

## 1. Introduction

**Figure 1:**
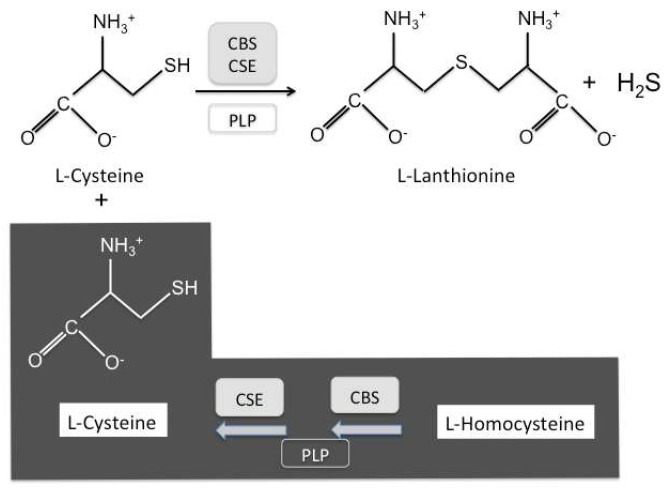
Biosynthesis of lanthionine from cysteine. Lanthionine (3,3′-thiodialanine) is a nonproteogenic amino acid, analog of cysteine, consisting of two alanine residues crosslinked on their β-carbon atoms by a thioether linkage. Full box indicates enzymes; open box indicates coenzymes. CBS, cystathionine-β-synthase; CSE, cystathionase. PLP, Pyridoxalphosphate. Lanthionine is stoichiometrically yielded as a stable byproduct of H_2_S biosynthesis. Dark background; complete transsulfuration pathway. Clear background; H_2_S biosynthesis by CBS and/or CSE independently.

Lanthionine is an unusual non-protein amino acid, naturally occurring as a byproduct of hydrogen sulfide (H_2_S) biosynthesis from cysteine in vivo [1,2]. This reaction is catalyzed by three enzyme systems in humans, cystathionine-β-synthase (CBS) or cystathionase (cystathionine-γ-lyase; CSE), and 3-mercaptopyruvate sulfurtransferase (MST), which does not generate lanthionine as a byproduct [3,4,5,6]. CBS and CSE are bifunctional enzymes in that they can catalyze either the complete two-step transsulfuration pathway, leading from homocysteine to cysteine, or each one, independently, the non-canonical H_2_S biosynthesis (Figure 1). Both CBS and CSE produce cystathionine, using cysteine and homocysteine as substrates, which from a kinetics point of view is predicted to be the most plausible H_2_S-generating reaction in vivo [3]. Alternatively, CBS and CSE may carry out H_2_S biosynthesis from cysteine yielding lanthionine, while CSE may also utilize homocysteine yielding homolanthionine [3]. Interestingly enough, both cystathionine and lanthionine have been found to be increased, as retention products, in uremia patients [7]. H_2_S is a gas, very difficult to quantitate because of its intrinsic volatility and its tendency to react with proteins [8]. Lanthionine has been considered a stable byproduct of H_2_S formation and hence a potential indicator of H_2_S biosynthesis, particularly in various biological systems [3,8]. However, lanthionine has been detected, in circulation, in uremic patients undergoing hemodialysis, which increases by about two orders of magnitude with respect to normal subjects [7], leading to the conclusion that renal function is a very important variable to take into account and, hence, the simple correlation between lanthionine and H_2_S production is an oversimplification [7]. In patients on dialysis, this compound can be considered among the retention products, responsible in general for uremic syndrome. The first biochemical evidence for this was that lanthionine is able to inhibit H_2_S formation in a hepatocarcinoma cell model (HepG2) [7]. Derangements of sulfur amino acid metabolism are very common in uremia, because of the high prevalence of hyperhomocysteinemia, a cardiovascular risk factor, which has been linked to the high cardiovascular mortality in these patients [7].

The zebrafish (*Danio rerio*) is a very versatile animal model for research on human diseases and potential therapies; in particular, it recently started to be employed to study uremic toxicity [9]. Fields of application include genetics, developmental biology, fertility, toxicology, and the mechanisms underlying various complications of human diseases. In this respect, for example, the zebrafish is considered a suitable model for investigating the microvascular complications of diabetes [10]. We were interested in exploring the effects of lanthionine toxicity and its underlying mechanisms. We studied the effects of lanthionine administered to zebrafish larvae, during early developmental stages, in view of its role as a potential uremic toxin with cardiovascular action.

## 2. Results

### 2.1. Effects of Lanthionine on Zebrafish Embryo Morphology, with a Focus on Cardiac Morphogenesis 

The aim of this part of our study was to observe whether lanthionine treatment was able to induce morphological changes in the early stages of developing zebrafish embryos. Zebrafish embryos were treated with various concentrations of lanthionine (0.3–2 μM). Moreover, since the two enzymes that catalyze the complete transsulfuration pathway are also independently responsible for H_2_S biosynthesis (Figure 1), it has been underscored that redox state is a crucial factor regulating the alternate switch between the two pathways [2,3,5]. Therefore, we also performed some experiments in the presence of glutathione (GSH: 100 μM), alone and in combination with lanthionine (lanthionine + GSH: 0.3 + 100 μM; 1 + 100 μM). 

**Figure 2:**
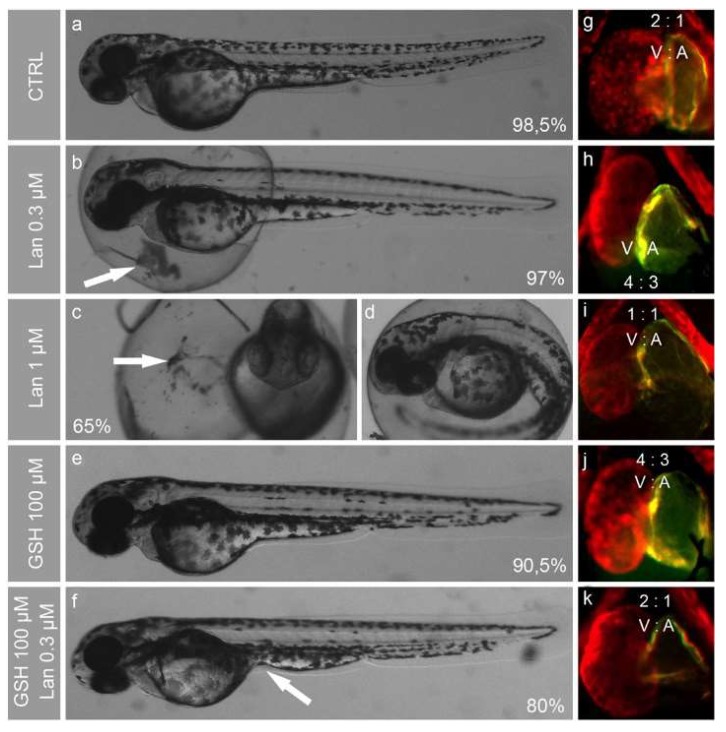
Developmental effects of lanthionine and/or GSH in zebrafish embryos at 72 hpf. (**a**–**f**) shows phenotypes of intact (**a**) and treated embryos (**b**,**c**,**e**,**f**), where the survival rates are indicated as percentage of still alive embryos at the end of treatment, compared to the initial number, in each group. Both frontal (**c**) and lateral (**d**) view is reported for 1 µM Lan treated embryos; panels (**a**,**b**,**d**–**f**) depict lateral views. Fluorescence microscope pictures (**g**–**k**) represents embryos hearts stained with anti-myosin antibodies (MF20 and S46) using IHC method (whole heart is stained in red and atrium in green). The size proportions of ventricle and atrium (V/A) are indicated in each heart image. Lan, lanthionine; CTRL, control (no treatment).

The effects of these compounds were monitored at different embryo developmental stages, with special regard to the morphogenesis of the cardiovascular system. In all experiments, lanthionine at 0.3 μM (the average circulating concentration detected in hemodialysis patient blood) and higher concentrations provoked yolk sac leaking (white arrows in Figure 2b,c) and induced delayed hatching (Figure 2b–d). At 72 h post-fertilization (hpf), when zebrafish heart formation is complete, we noticed a dose-dependent lanthionine effect onto the ventricle/atrium size ratio, evaluated from the 2D imaging, which, in untreated embryos, corresponded to 2:1 (Figure 2g). In embryos treated with 0.3 μM lanthionine, the atrium is enlarged by 50% (Figure 2h); 1 μM lanthionine induces the formation of an enlarged heart and alterations of its proportions, resulting in a ventricle and atrium of equal size (Figure 2i). Interestingly, 100 μM GSH led to 50% enlarged atrium (Figure 2j), and did not induce any other evident phenotypical alterations (Figure 2e); meanwhile, 100 μM GSH plus 0.3 μM lanthionine treatment resulted in heart chamber size proportions comparable to those of untreated embryos (Figure 2k). However, 30% of embryos in this experimental group showed yolk sac swelling or deformation (white arrow in Figure 2f). Embryos treated with 2 μM lanthionine displayed only 20% survival rate at 48 hpf, and, at 96 hpf, no individual survived. 

### 2.2. Effects of Lanthionine and GSH on Zebrafish Heart Rate

**Figure 3:**
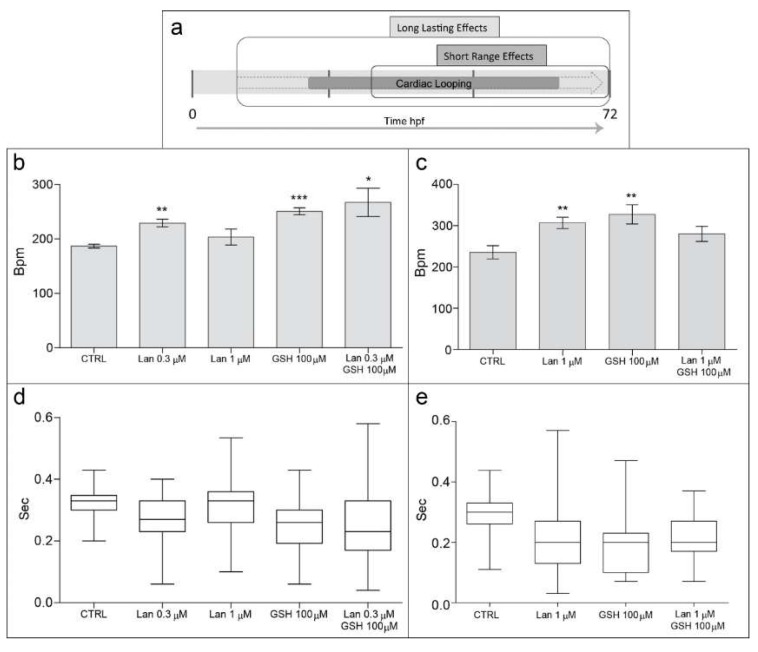
Effects of lanthionine and/or GSH on heart rate in zebrafish embryos at 72 hpf. (**a**) Represents duration of the two experimental sets, producing long lasting effects (~60 h, (**b**,**d**)) and short range effects (~40 h, **c** and **e**), respectively; unit of time is in hours; the bolder and semisolid arrow indicates the whole duration of zebrafish heart development, where cardiac looping phase is indicated by a solid symbol within the arrow. Heart beat frequency (**b**,**c**) shows the average number of heart beats per minute (bpm). The heart rhythm indicates the time interval, in seconds, between two contiguous heart beats (**d**,**e**). Treated embryos were compared to untreated controls. In (**b**,**c**); * *p* < 0.05, ** *p* < 0.01, *** *p* < 0.001, according to Student’s *t-*test. In (**d**,**e**) data are represented as box and whisker plot (mean difference *p* < 0.0001 according to one-way ANOVA test; variance difference, *p* < 0.0001 according to Bartlett’s test). Lan, lanthionine; CTRL, control (no treatment).

In order to infer a functional meaning of the above morphological changes, we evaluated the effects of lanthionine, in the presence or in the absence of GSH, during hearth organogenesis, according to the timing depicted in Figure 3a, on parameters of heart muscle contraction, analysis of heart rate indicators, composed of (i) heart rate (beat frequency) and (ii) heart rate variability (rhythm regularity), as reported in Figure 3b–e.

Handed positioning and cardiac looping, breaking cardiac symmetry during heart development, are crucial in order to both improve organ packaging in a limited cavity, and to insure proper heart function [11]. Consequently, the earliness of the exposure to a toxin, as well as its duration, is important to determine the extent of a functional alteration. In general, it can be postulated that, in relation to cardiac looping, an earlier and more prolonged exposure to a toxin will exert more drastic and long lasting functional effects, by affecting more profoundly organogenesis, while late and shorter treatments are expected to result in a more benign effect (see scheme in Figure 3a). 

Heart beat frequency, a standard parameter of toxicity that reflects the average number of heart beats per minute, was significantly influenced by all treatments except, apparently, by lanthionine at the higher concentration employed (Figure 3b). Paradoxically, the functional effect on heart frequency of high dosage of lanthionine may appear somewhat inconsistent with the pronounced cardiac teratogenesis, as it appears in Figure 2i. However, when lanthionine was administered at higher dose early during development, thus allowing it to act for a more extended period (i.e., long-lasting effects), heart rhythm changes could be observed (Figure 3c). In other words, data support the postulated crucial importance of lanthionine exposure duration in determining the severity of effects on cardiac morphology. 

We also measured, for every treatment heart rhythm, i.e., the variability of time length between two consecutive heart beats, which provided us with a more comprehensive evaluation of the effects of these treatments on cardiac function (Figure 3d,e). In this respect, although 1 μM lanthionine (short range effect) did not alter heart beat frequency significantly (Figure 3b), it clearly appeared to influence heart rhythm, which was significantly irregular (Figure 3d). These results indicate an increased propensity of lanthionine to induce a dose-dependent susceptibility to arrhythmia compared to control. The long-lasting GSH treatment was applied in order to encompass the entire period of cardiac looping [12] (see Figure 3a). The relevant results, of GSH on heart rhythm (Figure 3d) showed that, when GSH was administered after the start of heart development (short lasting effect), heart rhythm resulted more similar to that of the control. Embryos treated during 60–64 h (long-lasting experiment) with 1 μM lanthionine showed a 45% increase in heart beat frequency and pronounced arrhythmia; meanwhile embryos administered with 1 μM lanthionine and with 100 μM GSH showed a heartbeat frequency 32% higher than the control, although not significantly different, and no arrhythmia was detected (Figure 3c,e, respectively). 

Although GSH at 100 μM concentration appeared to counteract the teratogenic effects of 0.3 μM lanthionine (Figure 2e,f,j,k), it was unable to correct propensity to arrhythmia in zebrafish embryos (Figure 3d). 

### 2.3. Lanthionine Induces Locomotor Alterations in Zebrafish Embryos 

**Figure 4:**
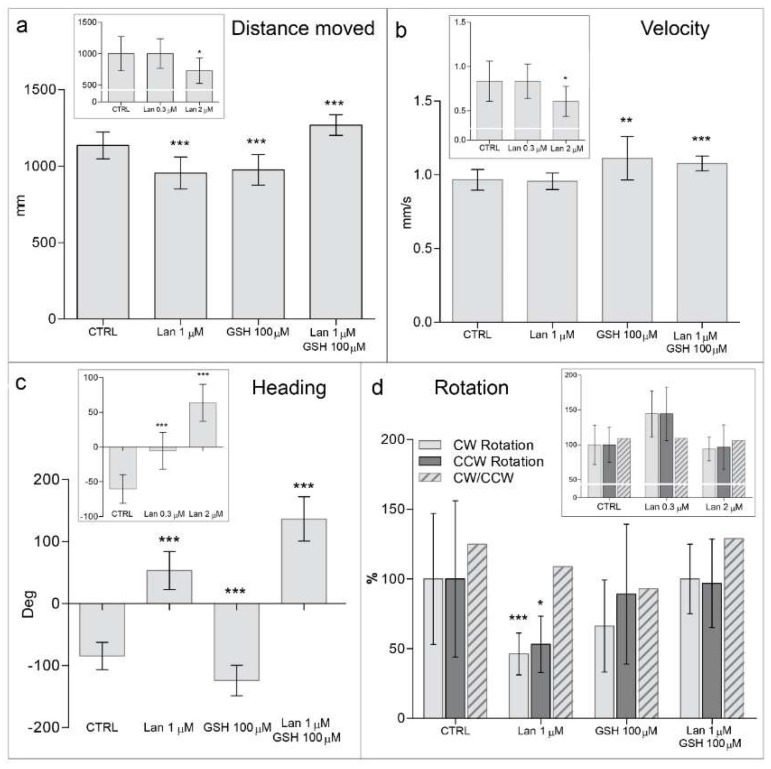
Behavioral effects of lanthionine on zebrafish embryos monitored by high-throughput tracking system. Diagrams **a**–**d** represent an analysis of locomotor activity of 72 and 105 hpf (diagram insets) zebrafish larvae under long lasting effects of lanthionine and/or GSH using high-throughput tracking system DanioVision^®^ (Noldus). (**a**) Shows embryos distance moved, (**b**) velocity, (**c**) heading (Deg; degrees), (**d**) clockwise (CW), counterclockwise (CCW) rotations and ratio between them. Treated embryos were compared to untreated controls. Data are depicted as mean ± SE (* *p* < 0.05, ** *p* < 0.01, *** *p* < 0.001, according to Student’s *t*-test). Lan, lanthionine; CTRL, control (no treatment).

Although delayed hatching cannot be considered a developmental staging marker for zebrafish [13], it may indicate that the embryos are less motile. This interpretation is also sustained by using high-throughput monitoring by means of DanioVision analysis (Figure 4). Quantification of locomotor activity in *D. rerio* larvae was accomplished by light-induced visual motor response [14,15]. The most representative parameters of the analysis, reflecting the average embryo behavior within the various treatment groups, are illustrated in Figure 4. Namely, two groups of locomotor alterations, the first dynamic (distance moved and velocity) and the second static (heading and rotation), after lanthionine and/or GSH treatment [16], were considered. In particular, rotation is related to the circular rotation of an embryo around its own body axis and heading is the inclination of an embryos head in degrees with respect to the water layer [16].

Thus, as for dynamic alterations, high-throughput tracking analysis showed lanthionine treatment induced a significant reduction of the distance that 72 hpf embryos covered in 20 min compared to control (Figure 4a). Embryos treated with higher lanthionine dosages, at 105 hpf, moved for shorter distances than untreated controls, likely because of the toxic effects exerted by lanthionine at these high concentrations (Figure 4a; inset). Lanthionine 0.3–1 μM did not affect the velocity of the embryos (Figure 4b). 

Static locomotor parameters (rotation, but especially heading) were also significantly altered by lanthionine treatment. Embryos were oriented toward water surface and rotated less than intact controls (Figure 4c,d, respectively). GSH treatment was in some instances able to prevent lanthionine-induced alterations of locomotor parameters, particularly with respect to rotation (Figure 4d). 

### 2.4. Molecular Alterations of Zebrafish Embryos Induced by Lanthionine: Enzymes, Structural Proteins, and Regulatory RNAs

In order to investigate at molecular level the alterations underlying the morphological and dysfunctional modifications of development that we observed above, we selected three groups of molecules: (a) enzymes and structural proteins; (b) microRNAs. 

One of the major structural alterations that could be detected upon lanthionine treatment was a change in the relative size of the heart chambers, accompanied by a number of relevant alterations of cardiac rhythm, particularly when lanthionine and GSH were contemporarily administered (Figure 2 and Figure 3). 

(a) Enzymes and structural proteins 

**Figure 5:**
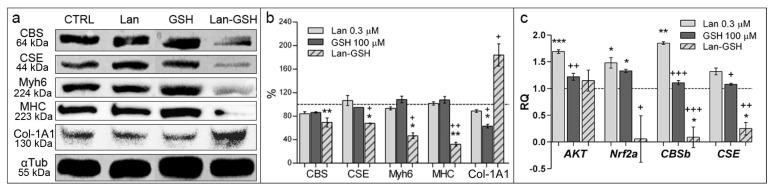
Molecular characterization of developmental alterations in zebrafish embryos. Analyses were performed in 105 hpf embryos treated with 0.3 µM lanthionine, 100 µM GSH or with both. (**a**) WB analysis of protein abundance in whole zebrafish embryos. 15 µg of total proteins were loaded; alpha tubulin (αTub); loading control; (**b**) Relative quantitation was expressed as percentage of band intensity, normalized to αTub and compared to the samples extracted from untreated control embryos (100%, dashed line). Protein bands were quantitated using ImageJ (National Institutes of Health). The columns represent the mean and error bars indicate the SD of band intensity from two independent experiments in triplicate; (**c**) Fold changes of *AKT*, *Nrf2a*, *CBSb* and *CSE* mRNA transcripts in 72 h of 0.3 μM lanthionine treatment (gray bars) in *D. rerio* embryos compared to untreated controls those indicated as dashed baseline. In (**b**) data are depicted as mean ± SEM. In both, (**b**) and (**c**), *p* value versus untreated controls; * *p* < 0.05, ** *p* < 0.01, *** *p* < 0.001, while *p* value versus lanthionine treated embryos; + *p* < 0.05, ++ *p* < 0.01, +++ *p* < 0.001 (according to Student’s *t-*test). Lan, lanthionine; CTRL, control (no treatment).

In order to help characterize, at molecular level, the observed developmental changes in zebrafish embryos upon treatments, we chose to analyze some proteins, including two myosins (MHC and Myh6), which are synthesized in the myocardial tissues, in zebrafish [17,18], a typical connective tissue protein (collagen type 1A1), and the transsulfuration enzymes CBS and CSE (Supplementary Materials, Table S1). In particular, CBS and CSE are bifunctional enzymes (see Figure 1) whose expression is widespread in several metabolically active tissues. Results showed that, upon treatment with lanthionine plus GSH, in particular, a number of alterations could be observed consisting in: a reduction of both CBS and CSE along with a significant decrease of both cardiac marker proteins, accompanied, in the same samples, with a parallel increase of collagen content (Figure 5a,b). It is worth noting that CBS and CSE have been previously found to be downregulated in uremia [19,20] and also that *Nrf2* and *AKT*, involved in the response to oxidative stress, are downregulated in renal disease development [21,22]. The qPCR analysis showed that lanthionine alone induced an increase of *CBSb* and *CSE* mRNA levels, which were not mirrored in the Western blot (WB) analysis (Figure 5). Conversely, GSH treatment together with lanthionine was able to convey lanthionine-induced decrease of expression of both *CBSb* and *CSE* (Figure 5c). qPCR analysis of *Nrf2a* showed that also the expression of this gene was affected by lanthionine and GSH in a similar fashion, downregulated by lanthionine plus GSH, while no changes could be detected on *AKT* transcript levels (Figure 5c). 

(b) MicroRNAs 

**Figure 6:**
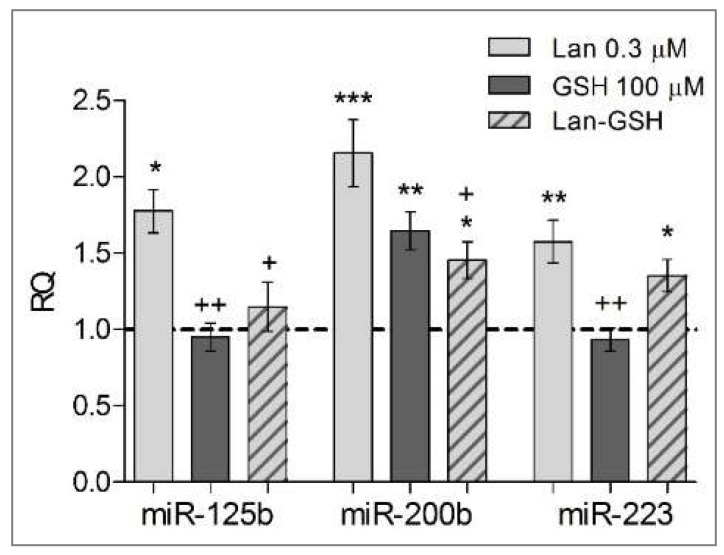
Effect of lanthionine and/or GSH on miRNAs. Fold changes in miRNA concentrations (miR-125b, miR-200b, miR-223) after 72 h of treatment in *D. rerio* embryos. Untreated controls; dashed baseline. * *p* < 0.05, ** *p* < 0.01, *** *p* < 0.001 versus untreated controls; + *p* < 0.05, ++ *p* < 0.01 versus lanthionine treated embryos (according to Student’s *t-*test). Lan, lanthionine.

We selected three microRNAs according to the following criteria (http://www.genome.jp/kegg/pathway.html; Supplementary Materials, Table S2): (i) they are found in both zebrafish and *Homo sapiens* (Supplementary Materials, Table S3); (ii) they regulate similar genes and functions in humans and in zebrafish to a substantial extent; (iii) their involvement in the pathogenesis of renal and/or heart disease has been put forward [23,24,25] (Supplementary Materials, Table S2). Three miRNA were found downregulated in zebrafish as the result of lanthionine (plus GSH) compared to untreated embryos (see Figure 6). Lanthionine, at concentrations comparable to those measured in vivo in uremia, induced a significant upregulation of miR-125b, miR-200b and miR-223, compared to control. Generally, miRNA levels were downregulated by GSH alone or when administered with lanthionine, in comparison to lanthionine alone (Figure 6).

## 3. Discussion

In this paper, we used zebrafish as a model to study the toxicity mechanisms of lanthionine, an unusual amino acid increased in uremic patients [7]. We demonstrated that lanthionine, at concentrations comparable to those actually measured in vivo in patients, is able to induce morphological alterations in zebrafish embryos at early developmental stages and also induced significant alterations of cardiac morphology (increased atrial size to the ventricle proportion) and function (heart beat frequency and tendency to arrhythmia are both increased). 

Recently zebrafish has been utilized to investigate the effects of uremic serum on zebrafish embryo survival [9]. The authors established that the uremic microenvironment significantly reduced survival rates on embryos versus control animals, challenged with normal serum. In addition, differences appeared to be due to complement activation, mainly related to protein-bound uremic retention compounds. A similar approach, using whole uremic serum, had been used to study the effects of uremic toxins on the osteogenic differentiation of human mesenchymal stem cells [23]. In both studies, indeed, it is quite likely that toxicity may be exerted by protein bound uremic toxins contained in the serum. In the present work we used a single compound, lanthionine, although at concentrations mimicking those which had been actually detected in patients. Then, although lanthionine is not expected to extensively bind to serum proteins [8], it is quite possible that some of the effects we observed on zebrafish embryos may be mediated, indeed, by an interaction of lanthionine with various proteins, such as in the case of homocysteine [24]. In order to identify some key molecular features in the mechanisms of lanthionine-induced alterations in zebrafish, which could be extrapolated to the human disease, we utilized two complementary approaches based on the theoretical selection of a few pathways, and relevant regulators, known to be altered in uremia, followed by their actual analysis in the zebrafish model system, upon treatment with lanthionine and/or GSH. 

Patients affected by chronic kidney disease, a growing population in the world, and especially those on hemodialysis, display a high mortality, whose causes are still to be fully understood. Cardiovascular accidents represent the highest cause of mortality in this population, accounting for about the 10% deaths per year, among hemodialysis patients; a toll ten times higher than that of healthy subjects [25,26]. For these reasons we mainly focused our work to the study of heart toxicity in zebrafish. However, we also employed the DanioVision technique to detect behavioral defects of the embryos, thus unravelling alterations in the maturation of the neurosensitive apparatus.

Many of the risk factors, affecting high cardiovascular mortality in renal disease, have been linked to uremic toxins or their derivatives [27]. Sulfur amino acid metabolism in particular has been found altered in many ways, with high plasma homocysteine, cystathionine, cysteine, *S*-adenosyl-*L*-homocysteine (the in vivo l-homocysteine precursor and a powerful inhibitor of methyltransferases) being present in uremia. In addition, low plasma and tissue levels of the endogenous gas H_2_S have been described, and this has been linked to a down-regulation of CSE, one of the main H_2_S-producing enzymes [19,20]. However, it is also possible that the observed increase in blood lanthionine could be linked, at least partially, to its increased intestinal production by the microbiota and subsequent absorption [28]. 

We have recently found that lanthionine is able to inhibit H_2_S formation, in a hepatocarcinoma cell line (HepG2) model, an effect likely to be due to lanthionine interference with *S*-adenosyl-l-methionine (AdoMet) action upon CBS, since, in the absence of this sulfonium compound, lanthionine inhibition of H_2_S release is abolished [7,8]. This could also contribute, at least in part, to the very high prevalence of hyperhomocysteinemia in uremia. The qPCR analysis we performed on zebrafish embryo extracts, showed that lanthionine alone induced an increase of *CBSb* and *CSE* mRNA levels, which were not exactly mirrored in the WB analysis (Figure 5). It should be pointed out, in this respect, that the kinetics of mRNA and protein increase may not necessarily be coincident and synchronous because of: (i) natural delay of protein biosynthesis, with respect to the increase in transcript levels; (ii) kinetics of transcript and protein turnovers (biosynthesis and degradation) may vary, and not necessarily be simultaneous, thus partly counterbalancing changes in the mRNA levels. As for *CBS* in particular, it is known that two *CBS* paralogues are present in zebrafish, as *CBSa* and *CBSb*. *CBSa* has been proven to be redundant [29] while in adult animals *CBSb* is expressed primarily in the kidney and muscle, with lower levels in the brain, gill, and heart [30] and its loss of function during embryogenesis, leads to increased levels of homocysteine [29]. In this work we only focused our qPCR analysis on the transcript encoding for the *CBSb*, while the anti-CBS antibody, used for WB was not paralog-specific. The latter consideration may contribute to explain the apparent gap between mRNA and protein levels. 

It should be mentioned that, beyond CBS and CSE, there is a third enzyme, MST, which is involved in H_2_S production. The reasons why in this manuscript we only focused on CBS and CSE are, in summary, the following: (i) MST, although capable of contributing to H_2_S production, is not a transsulfuration enzyme. More importantly, there is no evidence, so far, that lanthionine—our major topic in the present work—is indeed involved, as a substrate or a product or an effector, in the reaction catalyzed by MST [31]. (ii) It has been reported that, despite a H_2_S reduction, MST expression is actually increased in CKD patients, at least in mononuclear cells [20]. This may indicate that the MST increase, in this disease, is not capable of counterbalancing the decrease of *CBS* and/or *CSE* expression described both in the patients [20] and in uremia animal models [19]. On the other hand, MST is a multifunctional enzyme, and one of its functions is devoted to detoxification of cyanide, which is transformed into thiocyanate [32,33,34]. Actually, thiocyanide levels were also increased in blood of hemodialysis patients [35] and, despite MST upregulation in uremia, cyanide levels were still found elevated, suggesting that MST is perhaps saturated by these substrates. Therefore, we concluded that CBS and CSE were the major enzymes worth investigating in a model, such as zebrafish, intended to study lanthionine as a uremic toxin.

Contractile proteins become expressed in myocardial specialized tissues, as cardiac development takes place in zebrafish embryos. We detected a decrease of both Myh6 and MHC contractile proteins in the embryos treated with lanthionine in combination with GSH (Figure 5a,b), which could be linked, indeed, with morphological defects and relevant heart dysfunctions detected in Figure 2 and Figure 3. Since both contractile proteins are involved in the development of myocardial and muscle pathologies (Supplementary Materials, Table S1), it can be hypothesized that lanthionine and GSH may interact determining a tendency to develop fibrosis in zebrafish embryos. This interpretation is also strengthened by the analysis of the pathways and functions involving the above proteins (Supplementary Materials, Tables S2 and S3). Interestingly, when embryos were treated with a combination of lanthionine and GSH (at low GSH dosage; up to 50 µM), we could observe a typical additive effect, while, as GSH concentrations increased in the medium, the effect could rather be consistent with a synergistic model of action. This may be explained by the fact that transsulfuration enzymes, particularly CBS, are influenced by the oxidative microenvironment in many ways, also including a variation in the concentration of GSH [36,37]. 

The observed effects of GSH may be related to the ability of this compound to activate CBS by glutathionylation (Cys346) or by modifying the redox microenvironment [36]. Changes in the redox state of the microenvironment, induced by GSH, appeared to weaken these toxic effects of lanthionine on embryo morphology and heart physiology. However, at molecular level, the effects of lanthionine were remarkably evident even in the presence of GSH and appeared to be influenced by GSH in a more complex way. A further additional allosteric regulatory mechanism may depend on conformational changes induced by formation of a redox active disulfide bond in human CBS, and reversal by DTT, which has been recently shown to occur [37]. 

MicroRNAs are a vast group of signaling small RNAs that shed new light on the ways gene expression is regulated and open up new perspectives on the pathophysiology of disease mechanisms where altered gene expression may be involved. Three miRNA were found to be downregulated in zebrafish as the result of lanthionine treatment; the analysis of the “KEGG” pathway map showed that these miRNAs are relevant to fundamental pathways involved in processes such as inflammation, oxidative stress, connective tissue/extracellular cell matrix interactions and cell junctions, cardiovascular functions (Supplementary Materials, Table S2. Gene targets of these miRNAs listed in Table S3 (Supplementary Materials) may represent prospective deregulated proteins waiting to be tested.

## 4. Materials and Methods 

### 4.1. Materials

All materials and reagents were of the best available grade. Codes for all reagents used are available in Table S4 (Supplementary Materials).

### 4.2. Maintenance of Zebrafish and Embryos Treatment

WT AB zebrafish strain (*Danio rerio*) were maintained at Biogem (Ministry project code No. 78-17) according to standard procedures on a 14 h light/10 h dark cycle at 28.5 °C, as previously described [38]. Embryos were obtained by natural spawning. All protocols for zebrafish handling and for experiments involving not-feeding larvae (less than 120 hpf) were carried out according to Italian and European regulations and in accordance with the principles of the “Three Rs” directive, last updated: 19 December 2016.

Preliminary experiments were performed to establish embryos survival rate and, subsequently, the optimal concentrations of lanthionine and GSH to use for treatments, either alone or combined. Embryos at 12 hpf were exposed to lanthionine (0.03–3 μM), GSH (50–5000 μM) and lanthionine + GSH (0.3 μM + 100 μM; 1 μM + 100 μM) during following 88 h (Figure S1, Supplementary Materials). The mode of interaction between lanthionine and GSH was analyzed according to Markovsky et al. [39] based on the algorithm developed by Chou [40].

To investigate the effect of lanthionine, GSH and lanthionine with GSH on to cardiovascular system during development, embryos at 12 and 32 hpf were incubated for 40–60 h in E3 medium (5 mM NaCl, 0.17 mM KCl, 0.33 mM CaCl_2_, 0.33 mM MgSO_4_, 5% Methylene Blue) with 0.3–2 μM lanthionine, 100 μM GSH and lanthionine + GSH (0.3 μM + 100 μM; 1 μM + 100 μM), up to 100–105 hpf, then fixed or used for proteins and RNA extraction as described below. 

### 4.3. DanioVision Analysis

Alterations of locomotor activity of *D. rerio* larvae, at the indicated times of development, were evaluated in response to treatments using high-throughput monitoring, according to DanioVision analysis, by light-induced visual motor response (20 min: 10 min in dark and 10 min in light) [14,15]. Embryos were placed individually in 96 multi-well plates with a flat bottom and 200 µL of E3 medium. Quantification was accomplished by DanioVision equipment (Noldus, Wageningen, The Netherlands). This consists of the evaluation of various parameters including: distance moved, velocity, movement, mobility, distance to point, heading, turn angle, angular velocity, meander and rotation. The system is optimized in order to eliminate variations due to different well location effects, when comparing groups of animals subject to various treatments. 

### 4.4. Protein Extraction

Proteins from 72 hpf *D. rerio* embryos were extracted using RIPA buffer (HiMedia, Mumbai, India) with protease inhibitors cocktail (Roche S.p.A., Milan, Italy). Protein concentrations were determined according to Bradford (Protein Assay Kit, Bio-Rad, Milan, Italy). Samples were stored at −20 °C in preparation for Western blot analysis.

### 4.5. Western Blot Analysis

Proteins were separated on SDS, 15% polyacrylamide gels and transferred to Trans-Blot^®^ Turbo PVDF membrane (Bio-Rad). Protein detection was performed using the following primary antibodies: anti-CBS, anti-CSE, anti-myosin heavy chain (MHC) [17] and anti-myosin 6 [18] (MF20 and S46, DSHB, Iowa, IA, USA), anti-collagen 1A1 (sc-59772, Santa Cruz Biotechnology, Santa Cruz, CA, USA) and loading control anti-α tubulin (Abcam, Cambridge, UK). Secondary antibodies anti-rabbit and anti-mouse (Immunoreagents Inc., Raleigh, NC, USA) were conjugated with horseradish peroxidase (HRP). Immunocomplex visualization was obtained by chemiluminescence, utilizing Immobilon Western Chemiluminescent HRP Substrate (Merck Millipore, Milan, Italy). Signal intensity was quantified with the ChemiDoc™ (Bio-Rad) with the Bio-Rad Quantity One^®^ software version 4.6.3.

### 4.6. RNA Extraction, Amplification, and Analysis 

#### 4.6.1. RNA Extraction 

mRNA and miRNA were extracted from zebrafish embryos using mirVanaTM PARISTM kit (ThermoFisher Scientific, Milan, Italy) according to the supplier’s protocol. RNA concentration was measured by means of NanoDrop UV/Vis micro-spectrophotometry (ND-1000; NanoDrop Technologies, Wilmington, DE, USA). Samples were stored at −80 °C.

#### 4.6.2. Quantitative Real-Time PCR (qPCR) Gene Expression Analysis 

cDNA was synthesized from 1 μg of total RNA. For reverse transcription, the QuantiTect^®^ Reverse transcription kit and gDNA wipeout (Qiagen, Hilden, Germany) were used according to the supplier’s protocol. Reactions were performed in Veriti^®^ 96-Well Thermal Cycler (Applied Biosystems, Foster City, CA, USA). cDNA concentration was measured by means of NanoDrop UV/Vis micro-spectrophotometry (ND-1000; NanoDrop Technologies, Wilmington, DE, USA). cDNA samples were stored at −20 °C. 

qPCR experiments were performed using 200 ng cDNA and Power SYBR™ Green PCR Master Mix (Thermo Fisher) and ViiaTM7 Thermalcycler (Applied Biosystems). Oligonucleotides to amplify *D. rerio*: *AKT*, *Nrf2a*, *CBSb*, *CSE*, *GAPDH* genes were ordered from Thermo Fisher, appropriate oligo sequences are indicated in Table S5. Amplification conditions were the following: 95 °C for 15 min, followed by 40 cycles of 94 °C for 15 s, annealing step was carried out at 50 °C for 60 s and 72 °C for 30 s, to determine the relative quantities of the genes of interest transcripts present in the various experimental conditions compared with baseline. All our real-time PCR experiments were performed using *GAPDH* housekeeping gene as an internal control. We used the ΔΔCt method described in “Users bulletin”, ABI PRISM 7700 Sequence Detection System 1997. The data were handled by Real-Time PCR System ViiaTM7 software. 

#### 4.6.3. MiRNA Quantitative Real-Time PCR 

Selected miRNAs were retro-transcribed with RT-TaqMan^®^ MicroRNA Assays: miR-125b, miR-200b, miR-225 (Applied Biosystems) on Veriti^®^ 96-Well Thermal Cycler (Applied Biosystems) under following conditions: 16 °C for 30 min, 42 °C for 30 min, 85 °C for 5 min. Subsequently, obtained cDNA were amplified using corresponding TaqMan^®^ MicroRNA Assays (Applied Biosystems) on ViiaTM7 Thermalcycler (Applied Biosystems) under following conditions: 50 °C for 2 min, 95 °C for 10 min and 95 °C for 15 s, 60 °C for 1 min followed by 40 cycles in *D. rerio* samples. Relative quantification was performed using the ΔΔCt method using U6 snRNA as housekeeping. Differential levels of each miRNA expressed were evaluated by ViiaTM7 software (Applied Biosystems).

### 4.7. Heart Rate Evaluation 

This technique was developed by Enrico D’Aniello and Alessandra Gentile. An evaluation of the heart beat frequency and rhythm (heart rate variability) is based on the analysis of recorded zebrafish heart beatings video. The program of the Open Source Physics “Tracker 4.11.0” (http://www.opensourcephysics.org) is able to calculate brightness intensity changes of selected points in the registered video. Thus, zebrafish embryos at 72 hpf were registered using Integrated Standalone Digital Camera with Full HD Live Video Output Leica IC80 HD and Leica Stereo Microscope M60. Then, videos were analyzed using “Tracker 4.11.0” program option “Trace Region RGB”. Obtained data at red channel (the most relevant to embryos heart color), where each peak corresponds to one heartbeat. We performed analysis of 1-min videos (10 in every experimental group). The frequency of heart beating was established, counting the number of peaks (heartbeats) per minute; the regularity (rhythm) of heart beatings was established by comparing a time interval between every peak. Obtained data were analyzed with Prism5 GraphPad software. High deviation points to the presence of arrhythmia.

### 4.8. Immunohistochemistry, Imaging, and Analysis

Zebrafish embryos at 72 hpf were fixed for 1.5 h with 1% formaldehyde in PBS and used for immunohistochemistry procedure as previously described [41,42]. Briefly, fixed embryos were permeabilized using PBS with 0.2% saponin (Sigma-Aldrich, Milan, Italy), blocked in permeabilization solution with 10% sheep serum (Sigma) and 2 mg/mL BSA (Sigma). In order to localize zebrafish ventricle and atrium, we used MF20 (5 µg/mL, DSHB) and S46 antibodies. The fluorescent staining was developed using 1:500 secondary antibodies IgG Alexa Fluor^®^ 488 conjugate and IgG Alexa Fluor^®^ 555 conjugate (Thermo Fisher Scientific, Waltham, MA, USA). For fluorescent imaging we used Zeiss Axio Imager M1 microscope equipped with an “Axiocam HR” digital camera. Obtained images were analyzed using program package ImageJ 1.50b (National Institutes of Health, Bethesda, MD, USA) in order to compare the heart chambers size alterations in different experimental groups of zebrafish embryos. 

### 4.9. Statistical Analysis

Unpaired, two tailed Student’s *t*-test was utilized to compare means, as appropriate (means were considered significantly different as *p* < 0.05). One-way ANOVA test was used for the analysis of alterations of heart rhythm whereas indicated (means were significantly different when *p* < 0.05). For the analysis of equal variances, according to Bartlett’s test, variances differ significantly when *p* < 0.05. Data are expressed as the means ± standard deviation (SD), except where otherwise specified. Results were analyzed with the statistics software GraphPad Prism Version 6.0a for Apple Macintosh (GraphPad Software, San Diego, CA, USA). When studying the behavioral effects of lanthionine on zebrafish embryos by high-throughput tracking system, statistical analyses were generated by the DanioVision (Noldus) software system. 

## 5. Conclusions

Uremic toxins are a vast group of compounds of diverse chemical nature, from small molecules including aromatic compounds, to amino acid-related derivatives and polypeptides, which are retained in uremia, and are responsible for a substantial part of renal disease symptoms. In this study, we utilized the zebrafish as a novel model system to investigate the effects of a prospective uremic toxin: lanthionine, a natural byproduct of H_2_S biosynthesis, has recently been isolated from the serum of hemodialysis patients. In the present study the field of applications of the zebrafish model was remarkably widened, in that it was possible to identify the heart as the main target tissue of lanthionine toxicity and get insight into its molecular mechanisms. In particular, the results support the interpretation that lanthionine is able to induce heart tissue fibrosis and trigger alterations of regulatory RNA molecules involved in cardiovascular and renal diseases. The importance of the redox microenvironment, as an important element in conditioning lanthionine-dependent toxicity, has also been assessed. Data obtained from in silico approaches support the hypothesis of future strategies of potential therapeutic relevance to hinder its metabolic effects.

## Supplementary Materials

Supplementary materials can be found at http://www.mdpi.com/1422-0067/19/5/1323/s1.

## Abbreviations

CBS	Cystathionine-β-synthase;
CSE	Cystathionase
PLP	Pyridoxalphosphate
H_2_S	Hydrogen sulfide
qPCR	Quantitative real-time PCR
Cys	Cysteine
AdoMet	*S*-adenosyl-l-methionine
Lan	Lanthionine
GSH	Glutathione
CTRL	Control
IHC	Immunohistochemistry
hpf	Hours post-fertilization
bpm	Beats per minute
deg	Degree
CW	Clockwise
CCW	Counterclockwise
