# Supplementary material for: Zebrafish, a Novel Model System to Study Uremic Toxins: The Case for the Sulfur Amino Acid Lanthionine
Source: Int J Mol Sci. 2018 Apr 29;19(5):1323. doi: 10.3390/ijms19051323 (PMC5983689; doi:10.3390/ijms19051323)
Supplement: Supplementary file 1 [file ijms-19-01323-s001.pdf]

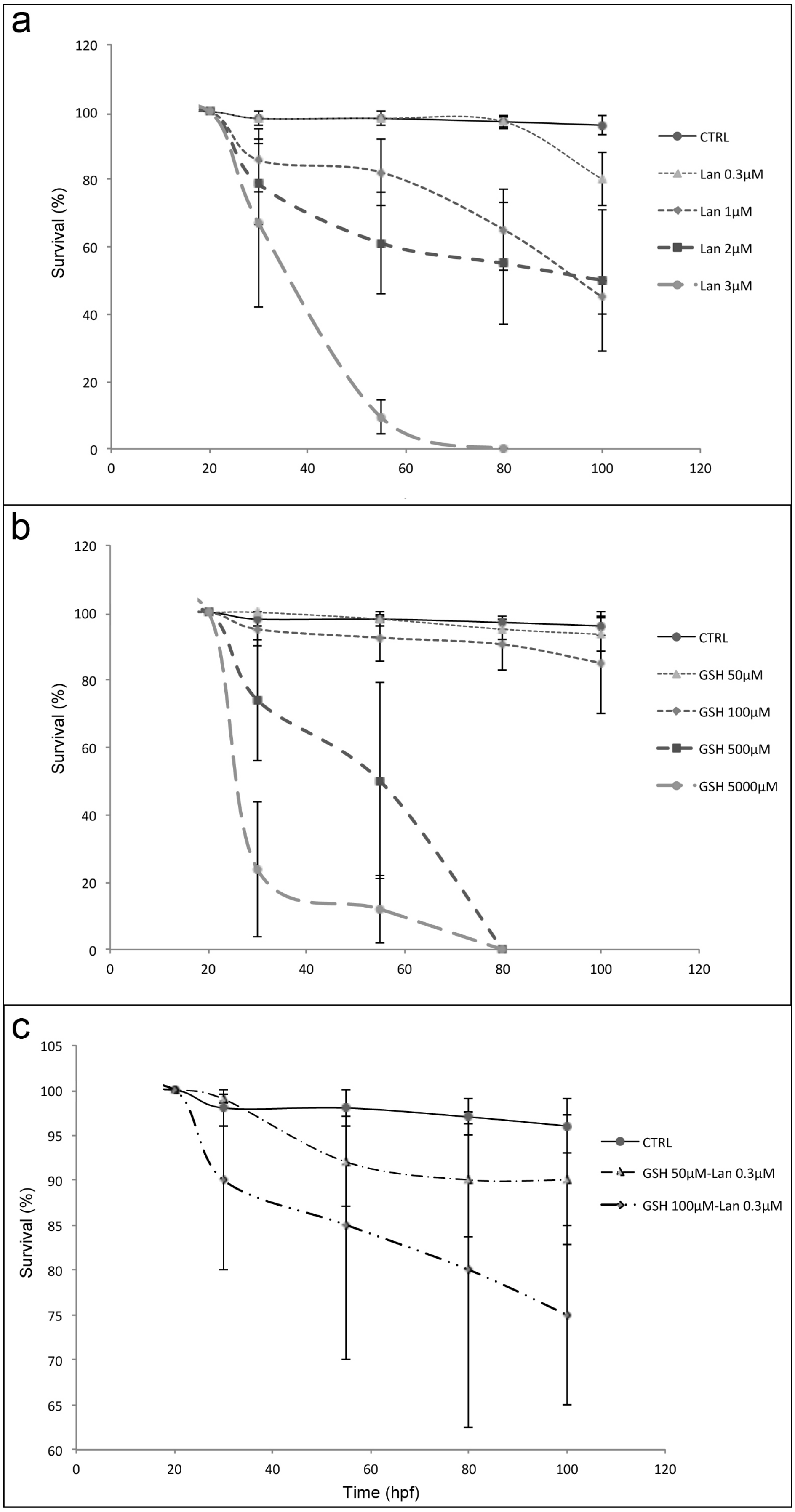

Figure S1. Survival rates of zebrafish embryos trated with lanthionine and/or GSH. Kinetics of survival rates of zebrafish embryos treated with lanthionine and GSH alone or in combination. Treatments started at 18 hpf; monitoring was accomplished, at the indicated times, within the 30-100 hpf interval. Results are reported in percentage of surviving animals at each time, compared to the initial number, within each observation group. (**a**) lanthionine at various concentrations; (**b)** GSH at various concentrations; **(c)** lanthionine in combination with GSH at various concentrations. Lan, lanthionine; CTRL, control (no treatment); hpf, hours post fertilization.

Table S1. *Functional involvement of the proteins affected by lanthionine and GSH administration in zebrafish.* Pathway nomenclature consists of a molecular network in terms of the KEGG (Kyoto Encyclopedia of Genes and Genomes) Orthology groups (*http://www.genome.jp/kegg/pathway.html*). In order to draw functional inferences about the individual proteins or on potential interactions among proteins, overall pathways were identified as generated according to the KEGG pathway map. Protein function and "Disease Ontology" is taken from zebrafish database Zfin (The Zebrafish Information Network; *https://zfin.org/*) .

PROTEIN	PATHWAY KEGG	FUNCTION	DISEASE ONTOLOGY
**CBS (CBSa AND CBSb PARALOGS)**	Cysteine and methionine metabolism<br>Biosynthesis of amino acids<br>Glycine, serine and threonine metabolism<br>Metabolic pathways	Cellular amino acid biosynthetic process<br>Cystathionine beta-synthase activity<br>Cysteine biosynthetic process<br>Cysteine biosynthetic process from serine<br>Cysteine biosynthetic process via cystathionine<br>Heme binding<br>Lyase activity<br>Pyridoxal phosphate binding	Homocystinuria
**CSE**	Oocyte meiosis<br>Selenocompound metabolism<br>Cysteine and methionine metabolism<br>Biosynthesis of amino acids<br>Glycine, serine and threonine metabolism<br>Metabolic pathways	'De novo' L-methionine biosynthetic process<br>Catalytic activity<br>Cystathionine gamma-lyase activity<br>Cysteine biosynthetic process via cystathionine<br>Lyase activity<br>Pyridoxal phosphate binding<br>Transsulfuration	Cystathioninuria
**Myh6**	Tight junction	Actin filament binding<br>ATP binding<br>Atrial cardiac myofibril assembly<br>Cardiac atrium development<br>Heart contraction<br>Heart morphogenesis<br>Motor activity<br>Myosin complex<br>Nucleotide binding	Atrial heart septal defect 3<br>Dilated cardiomyopathy 1EE<br>Hypertrophic cardiomyopathy 14
**MHC**	Vascular smooth muscle contraction<br>Adrenergic signaling in cardiomyocytes<br>Cellular senescence<br>Endocytosis<br>Cardiac muscle contraction<br>Cell adhesion molecules (CAMs)<br>Intestinal immune network for IgA production<br>Phagosome	Cardiac, skeletal and jaw muscle differentiation<br>Myosin-dependent functions<br>Myosin motor domain	Dilated cardiomyopathy 1S<br>Left ventricular noncompaction 5<br>Distal muscular dystrophy<br>Hypertrophic cardiomyopathy 1<br>Scapuloperoneal myopathy
**COL-1A1**	ECM-receptor interaction<br>AGE-RAGE signaling pathway in diabetic complications<br>Focal adhesion	Collagen trimer formation and cytoplasm formation<br>Extracellular matrix structural constituent<br>Response to mechanical stimulus<br>Fin development, morphogenesis and regeneration<br>Pectoral fin development<br>Regulation of ossification	Osteogenesis imperfecta type1-4<br>Osteoporosis<br>Type I Ehlers-Danlos syndrome

Table S2. *KEGG pathways in D. rerio involving miRNA-125b, miRNA-200b and miRNA-223.* Target genes for miRNA-125b, miRNA-200b and miRNA-223 were predicted using Target Scan Fish Release 6.2 (*http://www.targetscan.org/*). Pathways involving gene tragets for miRNA-125b, miRNA-200b and miRNA-223 as according to miRSystem software (ver. 20160513-miRNAsystem.cgm.ntu.edu.tw). In order to draw inferences on potential functional interactions between miRNA and their gene targets, pathways identified are listed according to the KEGG (Kyoto Encyclopedia of Genes and Genomes) pathway map. Relevant nomenclature consists of a molecular network in terms of the KEGG Orthology (KO) groups. miRNA genes targets analysis includes genes selected according to the relevance to processes involved renal failure deseases.

	PATHWAY	miR-125b	miR-200b	miR-223
ECM FORMATION AND CELL JUNCTION	**ECM-RECEPTOR INTERACTION**	*THBS1, ITGA9*	*DAG1, COL4A1, COL4A2, COL4A6, FN1, ITGA9, ITGB8, LAMC1*	*DAG1, COL1A2, COL4A1*
	**CELL ADHESION MOLECULES (CAMS)**	*CNTNAP2, ITGA9*	*CNTNAP2, SDC2, ITGA9, ITGB8*	*CNTNAP2, SDC2*
	**ADHERENS JUNCTION**		*MAPK1, CTNNA2, RAC1*	*IGF1, MAPK1, SMAD4*
	**GAP JUNCTION**	*PRKG1, MAP3K2*	*ADRB1, MAPK1, PRKG1, ADCY7, ITPR2, MAP2K1, MAPK7, PDGFB, PRKCA*	*ADRB1, KRAS, MAP2K5, MAPK1, PRKG1, ADCY7*
	**TIGHT JUNCTION**	*MAP2K7*	*MAPK1, MAPK10, MAP3K1, MAPK9, RAC1, PRKAG2, MAP2K7*	*MAPK1, PPP2CA, PRKCI, MAPK10, MAP3K1*
	**FOCAL ADHESION**	*PIK3R3, THBS1, ITGA9*	*COL4A1, MAPK1, PIK3R3, MAPK10, PAK7, COL4A2, COL4A6, FN1, ITGA9, ITGB8, LAMC1, MAP2K1, MAPK9, PDGFB, PRKCA, RAC1, RAP1B, BCL2*	*COL1A2, COL4A1, IGF1, MAPK1, VEGFAB, PIK3R3, MAPK10, PAK7*
	**REGULATION OF ACTIN CYTOSKELETON**	*PIK3R3, FGFR2, ITGA9*	*MAPK1, PIK3R3, PAK7, FGFR2, FN1, ITGA9, ITGB8, MAP2K1, PDGFB, RAC1*	*KRAS, MAPK1, PIK3R3, PAK7*
METABOLISM	**GLUTATHIONE METABOLISM**	*TXNDC12*	*RRM2*	
	**CYSTEINE AND METHIONINE METABOLISM**		*ENOPH1*	*ENOPH1*
	**ADIPOCYTOKINE SIGNALING PATHWAY**	*IRS1*	*MAPK1, TNFRSF1B, MAPK10, MAPK9, IRS1, PRKAG2, IKBKB, PRKCQ*	*MAPK1, TNFRSF1B, MAPK10*
	**CHOLESTEROL METABOLISM**	*LDLR*	*RAP1B*	*LDLR*
	**METABOLIC PATHWAYS**	*PLCG1*	*ENOPH1, IPPK, PLCG1, RRM2, PI4KB*	*ENOPH1, IPPK, PI4K2A*
FUNDAMENTAL REGULATORY/ SIGNAL TRANSDUCTION PATHWAYS	**MAPK SIGNALING PATHWAY**	*CACNA1C, MAP3K3, FGFR2, TP53, MAP2K7, MAP3K2, RPS6KA4*	*MAPK1, MAPK10, CACNA1C, CACNB1, MAP3K1, MAP3K3, FGFR2, MAP2K1, MAPK7, MAPK9, PDGFB, PRKCA, RAC1, RAP1B, TP53, IKBKB, MAP2K7, RPS6KA4, RPS6KA5*	*IGF1, KRAS, MAP2K5, MAPK1, VEGFAB, MAPK10, CACNA1C, CACNB1, MAP3K1, MAP3K3*
	**UBIQUITIN MEDIATED PROTEOLYSIS**	*UBE2R2, SMURF2*	*MAP3K1, UBE2J1, UBE2R2, SMURF2, SMURF1*	*MAP3K1, UBE2J1, UBE2R2, UBE4A*
	**NOTCH SIGNALING PATHWAY**	*NOTCH3*	*KAT2B*	*NOTCH3*
	**WNT SIGNALING PATHWAY**	*TP53*	*MAPK1, MAPK10, MAPK9, PRKCA, RAC1, TP53*	*MAPK1, SMAD4, MAPK10*
	**MTOR SIGNALING PATHWAY**	*PIK3R3, IRS1*	*MAPK1, PIK3R3, MAP2K1, PRKCA, IRS1, IKBKB*	*IGF1, KRAS, MAPK1, PIK3R3*
	**PHOSPHATIDYLINOSITOL SIGNALING SYSTEM**	*PIK3R3, PLCG1*	*PIK3R3, IPPK, ITPR2, PRKCA, PLCG1, PI4KB*	*PIK3R3, IPPK, PI4K2A*
	**INOSITOL PHOSPHATE METABOLISM, PI4KB**	*PLCG1*	*IPPK, PLCG1*	*IPPK, PI4K2A*
	**FOXO SIGNALING PATHWAY**	*PIK3R3, IRS1*	*MAPK1, PIK3R3, MAPK10, MAP2K1, MAPK9, BCL2, CDKN1B, IRS1, PRKAG2, IKBKB, IL10*	*IGF1, KRAS, MAPK1, SMAD4, PIK3R3, MAPK10*

	**GNRH SIGNALING PATHWAY**	*CACNA1C, MAP3K3, MAP2K7, MAP3K2*	*MAPK1, MAPK10, ADCY7, CACNA1C, MAP3K1, MAP3K3, ITPR2, MAP2K1, MAP2K1, MAPK7, MAPK9, PRKCA, MAP2K7*	*KRAS, MAPK1, MAPK10, ADCY7, CACNA1C, MAP3K1, MAP3K3*
	**CALCIUM SIGNALING PATHWAY**	*CACNA1C, PLCG1*	*ADRB1, ADCY7, CACNA1C, ITPR2, PRKCA, PLCG1, ATP2B2*	*ADRB1, ADCY7, CACNA1C*
RENAL AND CARDIOVASCULAR FUNCTION	**CARDIAC MUSCLE CONTRACTION**	*CACNA1C*	*ATP1B4, CACNA1C, CACNB1*	*ATP1B4, CACNA1C, CACNB1*
	**VASCULAR SMOOTH MUSCLE CONTRACTION**		*MAPK1, PRKG1, PERP, ADCY7, ITPR2, MAP2K1, PRKCA, PRKCQ*	*MAPK1, PRKG1, PERP, ADCY7*
	**HYPERTROPHIC CARDIOMYOPATHY (HCM)**	*CACNA1C*	*DAG1, CACNA1C*	*DAG1, CACNA1C*
	**VEGF SIGNALING PATHWAY**	*PIK3R3, PLCG1*	*MAPK1, PIK3R3, MAP2K1, PRKCA, RAC1, PLCG1*	*KRAS, MAPK1, VEGFAB, PIK3R3*
	**ADRENERGIC SIGNALING IN CARDIOMYOCYTES**	*CACNA1C*	*ADRB1, MAPK1, PPP2CA, ATP1B4, ADCY7, CACNA1C, CACNB1, PRKCA, BCL2, ATP2B2, RPS6KA5*	*ADRB1, MAPK1, PPP2CA, ATP1B4, ADCY7, CACNA1C, CACNB1*
	**APELIN SIGNALING PATHWAY**	*NOTCH3*	*MAPK1, ADCY7, ITPR2, PRKAG2*	*KRAS, MAPK1, SMAD4, ADCY7, NOTCH3*
	**AGE-RAGE SIGNALING PATHWAY IN DIABETIC COMPLICATIONS**	*PIK3R3, PLCG1, EDN1*	*COL4A1, MAPK1, PIK3R3, MAPK10, COL4A2, COL4A6, FN1, MAPK9, PRKCA, RAC1, BCL2, CDKN1B, PLCG1*	*COL1A2, COL4A1, KRAS, MAPK1, SMAD4, VEGFAB, PIK3R3, MAPK10*
	**INSULIN SIGNALING PATHWAY**	*IRS1*	*MAPK1, MAP2K1, MAPK9, IRS1, PRKAG2, PRKAR2B, IKBKB*	*MAPK1*
CANCER	**P53 SIGNALING PATHWAY**	*THBS1, CDK6, TP53*	*E2F1, IGFBP3, CCNE2, CDK6, TP53, RRM2*	*IGF1, E2F1, IGFBP3, PERP*
IMMUNE SYSTEM	**CYTOKINE-CYTOKINE RECEPTOR INTERACTION**	*IL2RB*	*IL2RB, IL6ST, TNFSF11, PDGFB, IL10*	*IL2RB, VEGFAB, VEGFB, TNFRSF1B, IL6ST, TNFSF11*
	**TGF-BETA SIGNALING PATHWAY**	*SMURF2, THBS1*	*MAPK1, PPP2CA, SMURF2, SMAD1, SMURF1*	*MAPK1, PPP2CA, SMAD4*
	**TOLL-LIKE RECEPTOR SIGNALING PATHWAY**	*PIK3R3, MAP2K7*	*MAPK1, PIK3R3, MAPK10, IRF5, MAP2K1, MAPK9, RAC1, IKBKB, IL10, MAP2K7*	*MAPK1, PIK3R3, MAPK10, IKBKE, IRF5*
	**NOD-LIKE RECEPTOR SIGNALING PATHWAY**	*TP53*	*MAPK1, MAPK10, FN1, ITPR2, MAPK9, BCL2, TP53, IKBKB, IL10*	*MAPK1, MAPK10, IKBKE*

Table S3. Target genes in *D. rerio*, regulated by at least two given miRNAs in over two analyzed pathways in human according to KEGG databease (see Table 2). A particular attention was payed to genes, regulated by 3 miRNAs simultaneously (shown in underlined bold: *CACNA1C, CNTNAP2, IL2RB, MAP3K3, PIK3R3, PRKG1, UBE2R2*).

miR-125b	miR-200b	miR-223
	ADCY7	*ADCY7*
	ADRB1	*ADRB1*
	ATP1B4	*ATP1B4*
CACNA1C	***CACNA1C***	***CACNA1C***
	CACNB1	*CACNB1*
CDK6	*CDK6*	
CNTNAP2	***CNTNAP2***	***CNTNAP2***
	COL4A1	*COL4A1*
	DAG1	*DAG1*
DYNC1LI2		*DYNC1LI2*
	E2F1	*E2F1*
	ENOPH1	*ENOPH1*
FGFR2	*FGFR2*	
	IGFBP3	*IGFBP3*
IL2RB	***IL2RB***	***IL2RB***
	IL6ST	*IL6ST*
	IPPK	*IPPK*
	IRF5	*IRF5*
IRS1	*IRS1*	
ITGA9	*ITGA9*	
KCNJ11	*KCNJ11*	
LDLR		*LDLR*
MAP2K7	*MAP2K7*	
	MAP3K1	*MAP3K1*
MAP3K3	***MAP3K3***	***MAP3K3***
	MAPK1	*MAPK1*
	MAPK10	*MAPK10*
NOTCH3		*NOTCH3*
	PAK7	*PAK7*
	PDCD6IP	*PDCD6IP*
PIK3R3	***PIK3R3***	***PIK3R3***
PLCG1	*PLCG1*	
	PPP2CA	*PPP2CA*
PRKG1	***PRKG1***	***PRKG1***
RPS6KA4	*RPS6KA4*	
	SDC2	*SDC2*
SMURF2	*SMURF2*	
	TNFSF11	*TNFSF11*
TP53	*TP53*	
	UBE2J1	*UBE2J1*
UBE2R2	***UBE2R2***	***UBE2R2***

Table S4. *Antibodies and reagents used.*

ANTIBODIES

**Antigene**	**Acronims**	**Code**	**Company**	**Citations**
Human, *D. rerio* Cystathionine β-Synthase	anti-CBS	ab96252	Abcam	
Human, *D. rerio* Cystathionine γ-Lyase	anti-CSE	ab136604	Abcam	
D. rerio Myosin, sarcomere (MHC)	MF20	AB2147781	DSHB	[17]
D. rerio Myosin heavy chain, slow developmental (sd-MyHC/ Myh6)	S46	AB528376	DSHB	[18]
Human, Collagen Type I	Col-1A1	sc-59772	Santa Cruz Biotechnology	
Human, zebrafish, anti-alpha Tubulin	αTub	ab15246	Abcam	
Horseradish peroxidase conjugated anti-rabbit	anti-rabbit	NC27606	Immunoreagents	
horseradish peroxidase conjugated anti-mouse	anti-mouse	NC	Immunoreagents	
IgG Alexa Fluor® 488 conjugate	anti-mouse	A-11001	Thermo Fisher	
IgG Alexa Fluor® 555 conjugate	anti-mouse	A-21422	Thermo Fisher	

REAGENTS

**Name of the reagent**	**Abbreviation**	**Code**	**Producer**
Dulbecco's Modified Eagle's Medium	DMEM	L0106-500	Microgem
Fetal Bovine Serum	FBS	S1810-500	Microgem
L-Glutamine		X0550-100	Microgem
Penicillin-Streptomycin Solution	Pen-strep	L0022-100	Microgem
L-Glutathione	GSH	G6013-5G	Sigma
DL-Cysteine	Cys	30089-100G	Sigma
Pyridoxine hydrochloride	B6	P6280-10G	Sigma
S-Adenosylmethionine	SAM	B9003S	BioLabs
DL-Lanthionine	Lan	L0010	TCI
RIPA buffer		TCL131	Himedia
Protease Inhibitor Cocktail Tablets	protease inhibitors	11836153001	Roche
Trans-Blot® Turbo™ Mini PVDF Transfer Packs	PVDF membrane	1704156	Bio-rad
Immobilon Western Chemiluminescent HRP Substrate		WBKLS0500	Merck
mirVana™ PARIS™ kit		AM1556	Thermo Fisher
QuantiTect® Reverse transcription kit		205313	Qiagen
Power SYBR™ Green PCR Master Mix		4367659	Thermo Fisher
RT-TaqMan® MicroRNA Assays			Applied Biosystems
Saponin		47036-50G	Sigma-Aldrich
Sheep Serum		S3772-5ML	Sigma
Bovine Serum Albumin	BSA	A2153-10G	Sigma

Table S5. *List of oligonucleotides used for qPCR experiments.*

Primer Name		Sequence (5′ → 3′)	Source	ID
Nrf2a	Forward	GAGCGGGAGAAATCACACAGAATG	[43]	NM_182889.1
	Reverse	CAGGAGCTGCATGCACTCATCG		
AKT	Forward	GCAAGATGTGGATCAGCTGGAG	[44]	NM_001281801.1
	Reverse	CCACAGTCTGGATGGCTTGGT		
CBSb	Forward	TTGACCAGTACCGCAATCCC	[30]	NM_001014345.2
	Reverse	CCTGCGACCAGCATGTCTAT		
CSE	Forward	CGTCTTTCAGTGGGTCTGGA	[30]	NM_212604.3
	Reverse	CACTGCTGTTCCTCATCCGT		
GAPDH	Forward	GATACACGGAGCACCAGGTT	[44]	NM_001115114.1
	Reverse	GCCATCAGGTCACATACACG		

Supplementary References

43. Rousseau, M.E.; Sant, K.E.; Borden, L.R.; Franks, D.G.; Hahn, M.E.; Timme-Laragy, A.R. Regulation of Ahr signaling by Nrf2 during development: Effects of Nrf2a deficiency on PCB126 embryotoxicity in zebrafish (Danio rerio). Aquat. Toxicol. 2015, 167, 157–171.
44. Li, L.; Huang, T.; Tian, C.; Xiao, Y.; Kou, S.; Zhou, X.; Liu, S.; Ye, X.; Li, X. The defensive effect of phellodendrine against AAPH-induced oxidative stress through regulating the AKT/NF-κB pathway in zebrafish embryos. Life Sci. 2016, 157, 97–106.
